# NK Cell–Monocyte Cross-talk Underlies NK Cell Activation in Severe COVID-19

**DOI:** 10.4049/jimmunol.2300731

**Published:** 2024-04-26

**Authors:** Madeline J. Lee, Izumi de los Rios Kobara, Trisha R. Barnard, Xariana Vales Torres, Nicole H. Tobin, Kathie G. Ferbas, Anne W. Rimoin, Otto O. Yang, Grace M. Aldrovandi, Aaron J. Wilk, Jennifer A. Fulcher, Catherine A. Blish

**Affiliations:** * Department of Medicine, Stanford University School of Medicine, Palo Alto, CA; † Stanford Immunology Program, Stanford University School of Medicine, Palo Alto, CA; ‡ Division of Infectious Diseases, Department of Pediatrics, David Geffen School of Medicine at UCLA, Los Angeles, CA; § Department of Epidemiology, Fielding School of Public Health, University of California, Los Angeles, Los Angeles, CA; ¶ Division of Infectious Diseases, Department of Medicine, David Geffen School of Medicine at UCLA, Los Angeles, CA; ‖ Stanford Medical Scientist Training Program, Stanford University School of Medicine, Palo Alto, CA; # Chan Zuckerberg Biohub, San Francisco, CA

## Abstract

NK cells in the peripheral blood of severe COVID-19 patients exhibit a unique profile characterized by activation and dysfunction. Previous studies have identified soluble factors, including type I IFN and TGF-β, that underlie this dysregulation. However, the role of cell–cell interactions in modulating NK cell function during COVID-19 remains unclear. To address this question, we combined cell–cell communication analysis on existing single-cell RNA sequencing data with in vitro primary cell coculture experiments to dissect the mechanisms underlying NK cell dysfunction in COVID-19. We found that NK cells are predicted to interact most strongly with monocytes and that this occurs via both soluble factors and direct interactions. To validate these findings, we performed in vitro cocultures in which NK cells from healthy human donors were incubated with monocytes from COVID-19^+^ or healthy donors. Coculture of healthy NK cells with monocytes from COVID-19 patients recapitulated aspects of the NK cell phenotype observed in severe COVID-19, including decreased expression of NKG2D, increased expression of activation markers, and increased proliferation. When these experiments were performed in a Transwell setting, we found that only CD56^bright^ CD16^−^ NK cells were activated in the presence of severe COVID-19 patient monocytes. O-link analysis of supernatants from Transwell cocultures revealed that cultures containing severe COVID-19 patient monocytes had significantly elevated levels of proinflammatory cytokines and chemokines, as well as TGF-β. Collectively, these results demonstrate that interactions between NK cells and monocytes in the peripheral blood of COVID-19 patients contribute to NK cell activation and dysfunction in severe COVID-19.

## Key Points

- scRNA-seq analysis predicts interactions between NK cells and monocytes in COVID-19.
- Monocytes from COVID-19 donors induce activation and proliferation in healthy NK cells.
- CD56^bright^ NK cells are activated by monocyte-derived soluble proinflammatory factors.

## Introduction

Natural killer cells are innate lymphocytes that are critical antiviral effectors. Because of their role in controlling acute viral infections, multiple studies have evaluated the role of NK cells in SARS-CoV-2 infection. Such studies revealed that NK cell phenotype and function are significantly altered by severe COVID-19; the peripheral NK cells of severe COVID-19 patients are highly activated and proliferative (1–6), with increased expression of cytotoxic molecules, Ki-67, and several surface markers of activation (3, 5, 7–9). However, these NK cells also have dysfunctional cytotoxic responses to both tumor target cells (1,2, 10,11) and SARS-CoV-2–infected target cells (10,11). Given that peripheral NK cells are thought to migrate to the lung during COVID-19 (12–14), these results suggest that the NK cells of severe COVID-19 patients may be incapable of mounting a successful antiviral response to SARS-CoV-2 infection.

Although the unique phenotype and dysfunctionality of NK cells in severe COVID-19 have been well characterized, the processes underlying these phenomena have not. Only one study has conducted in vitro mechanistic experiments to identify a possible cause of NK cell dysfunction: Witkowski et al. (10) identified serum-derived TGF-β as a suppressor of NK cell functionality in severe COVID-19 patients. However, this study did not identify the source of serum TGF-β. Additionally, given the high degree of complexity within the immune system, there are likely other causes of NK cell dysfunction in COVID-19 that have thus far remained unexplored. One such mechanism may be the myriad of interactions between NK cells and other peripheral immune cells. NK cells are known to interact with CD4 and CD8 T cells, dendritic cells, neutrophils, and macrophages/monocytes (15), which can prime NK cell cytotoxicity or induce tolerance. Previous work by our laboratory suggested the potential for NK cell–monocyte cross-talk in severe COVID-19 through the expression of ligands for NK cell–activating receptors on the monocytes of these patients (3). Cross-talk between NK cells and monocytes plays a role in regulating the NK cell response to other infections, including HIV-1 (16,17), mouse (18) and human CMV (19), and malaria (20), through mechanisms including secretion of NK cell–regulating cytokines by monocytes.

In this study, we used a combination of computational and in vitro methods to dissect the interactions between NK cells and monocytes in severe COVID-19. We used primary NK cells and monocytes from a large cohort of COVID-19 patients to demonstrate that coculture of healthy NK cells with monocytes from severe COVID-19 donors can partially recapitulate the activated phenotype observed in the NK cells from COVID-19 patients. We then interrogated the mechanisms by which this activation occurs by performing NK cell–monocyte cocultures in a Transwell setting and using O-link to analyze the cytokines present in this system. Collectively, our work identifies monocytes as a driver of NK cell activation in severe COVID-19 and reveals interactions between NK cells and monocytes that may underlie this process.

## Materials and Methods

### Cohort

Samples from hospitalized COVID-19 patients were obtained from an observational cohort study of hospitalized COVID-19 patients at UCLA. All participants signed informed consent to participate, and the study was approved by the UCLA Institutional Review Board (approval no. 20–000473). Patients were recruited from two UCLA Health hospitals in Los Angeles, CA. Inclusion criteria included hospitalization for COVID-19, age greater than 18, and confirmed positive SARS-CoV-2 RT-PCR within 72 h of admission. Exclusion criteria included pregnancy, hemoglobin less than 8 g/dl, inability to provide informed consent, or solid organ transplant. Upon enrollment, blood samples, nasopharyngeal swab, and saliva were collected throughout hospitalization up to 6 wk. Demographic and clinical data, including therapeutics, were collected from the electronic medical record. Clinical severity was scored using the NIAID 8-point ordinal scale (21): 1, not hospitalized and no limitations; 2, not hospitalized but with limitations; 3, hospitalized, no supplemental oxygen or ongoing medical care; 4, hospitalized no supplemental oxygen but with ongoing medical care; 5, hospitalized with supplemental oxygen; 6, hospitalized with noninvasive ventilation or high-flow oxygen; 7, hospitalized with invasive mechanical ventilation or extracorporeal membrane oxygenation (ECMO); and 8, death. For this study, mild COVID-19 included ordinal scale 3 or 4, moderate COVID-19 included ordinal scale 5, and severe COVID-19 included ordinal scale 6 or 7. The samples included in this study were collected from April 2020 through February 2021.

### Monocyte isolation

Cryopreserved PBMCs from COVID-19–positive and healthy donors were thawed at 37°C and washed with RPMI 1640 supplemented with 10% FBS (RP10) to remove freezing medium. Cells from each donor were magnetically fractionated into CD14^+^ and CD14^−^ populations using the Miltenyi MACS human CD14+ microbead isolation kit (Miltenyi, catalog no. 130-050-201) according to the manufacturer’s instructions. CD14^+^ cells were then set aside in an incubator (37°C, 5% CO_2_) until the start of coculture, whereas CD14^−^ cells were used for subsequent NK cell isolation.

### NK cell isolation and activation

NK cells were isolated from CD14^−^ cells from healthy donors and COVID-19 patients using the Miltenyi MACS human NK cell isolation kit (Miltenyi, catalog no. 130-092-657) according to the manufacturer’s instructions. 10% of NK cells from healthy and COVID-19–positive donors were set aside for phenotyping by flow cytometry. NK cells from two healthy donors (MJL01 and MJL03) were set aside for monocyte coculture assays. The remaining NK cells were transferred to a round-bottom 96-well plate and resuspended in complete RPMI 1640 supplemented with 25 ng/ml (250 IU/ml) rhIL-2 (R&D Systems, catalog no. 202-IL-010) and then placed in a 37°C CO_2_ incubator for 12–16 h. After incubation, the cells were washed twice to remove IL-2, counted, and resuspended in fresh RP10 before being transferred to BSL3 facilities for killing assays.

### NK cell phenotyping

As described above, samples of NK cells from healthy donors and COVID-19 patients were taken for phenotyping by flow cytometry. NK cells were washed in PBS and stained with eFluor 780 fixable viability dye (eBioscience, catalog no. 65-0865-14) for 20 min. The cells were then washed in FACS buffer (PBS supplemented with 2% FBS) and stained for 30 min at room temperature with a panel of Abs against surface Ags. Stained NK cells were washed, fixed for 15 min in 4% paraformaldehyde (EIS, catalog no. 15710), and permeabilized (BD Biosciences, catalog no. 340973). Permeabilized cells were stained with a panel of Abs against intracellular proteins, then washed, and analyzed on a Cytek Aurora spectral cytometer.

### Cell lines

A549-ACE2 were a gift from Ralf Bartenschlager and were confirmed to be mycoplasma-free. A549-ACE2 cells were maintained in DMEM supplemented with 10% FBS and passaged every 2–3 d. The cell cultures were discarded and new cells were thawed after 25 passages.

### Infection of A549-ACE2 with SARS-CoV-2

The day prior to infection, A549-ACE2 cells were seeded at a density of 100,000 cells/well in a 12-well plate. On the day of infection, the cells were brought into the BSL3 and washed once with PBS to remove excess serum. PBS was then removed, and mNeon Green SARS-CoV-2 was added in DMEM supplemented with 2% FBS (D2) at an multiplicity of infection of 0.5 (final volume of 150 μl/well). The plate was rocked for 1 h at 37°C, after which time the virus was washed off with PBS and 0.5 ml of D2 was added to each well. The plates were placed back in an incubator for 48 h before being harvested for use in killing assays.

### Flow cytometry–based killing assay

The morning of the killing assay, IL-2–activated NK cells were counted and brought into the BSL3. SARS-CoV-2–infected A549-ACE2s were washed with PBS, harvested using TrypLE, and counted before being resuspended in fresh RP10. Target cells and NK cells were plated in V-bottom 96-well plates at an effector:target ratio of 10:1. The plate containing target cells and NK cells was spun down for 1 min at 1,000 rpm to bring cells together and then placed in the 37°C incubator for 3 h. After 3 h, the cells were washed with PBS and stained with eFluor 780 fixable viability dye for 25 min. The cells were then washed in PBS and fixed for 30 min in 4% paraformaldehyde before being transferred to fresh tubes, decontaminated, and removed from the BSL3 and analyzed on a Cytek Aurora spectral cytometer.

### Allogeneic NK cell/monocyte coculture

Purified CD14^+^ cells in RP10 were added to a round-bottom 96-well plate or the bottom of a 24-well Transwell plate for a final concentration of 1.5 × 10^6^ CD14+ cells/ml. Two replicates were plated for each monocyte donor; each of these replicates then received NK cells from one of two healthy donors (MJL01 or MJL03) at a final concentration of 0.75 × 10^6^ NK cells/ml. These NK cells were not preactivated with IL-2 or otherwise treated prior to coculture. NK cells were added directly to monocytes in round-bottom 96-well plates or to the top of a 0.4-μm Transwell insert in Transwell plates. Both culture systems had a monocyte:NK ratio of 2:1, and the final concentration of cells in the media was kept consistent between the two culture systems. Once the cells had been added, the 96-well culture plates were spun down for 1 min at 1,000 rpm to bring the cells together. Spun-down 96-well plates and Transwell culture plates were then placed in a 37°C incubator for 2 h. After 2 h, NK cells from the Transwell inserts were collected and transferred to a fresh 96-well plate. NK cells from all cultures were then stained for flow cytometry and analyzed in the manner described above (under “NK cell phenotyping”). The Transwell and direct cultures for each donor were performed simultaneously to minimize batch effects.

### O-link

After the NK cells were harvested from the Transwell cultures, the remaining Transwell culture supernatants were saved for O-link analysis. The supernatants were collected in microcentrifuge tubes and centrifuged to remove any cells and cell debris in the sample. Once clarified, the supernatants were transferred to fresh tubes and frozen at −80°C until analyzed. The samples did not undergo any freeze–thaw cycles other than when they were thawed for final analysis. O-link was performed in technical duplicate according to the manufacturer’s instructions using the 92-analyte inflammation panel from O-link (22).

### Scriabin analysis

Small conditional RNA sequencing (scRNA-seq) data from Wilk et al. (3) was first passed through a denoising algorithm (Adaptively Thresholded Low-rank Approximation - “ALRA”), which uses low-rank matrix approximation to impute expression levels of lowly expressed genes, thereby partially alleviating the sparsity of the gene expression matrix (23). Scriabin was then used to identify sets of highly coexpressed ligand–receptor pairs and group them into interaction programs (IPs) whose expression could be compared between sample conditions and cell types. More detail regarding the detailed methodology underpinning *Scriabin* can be found in the article describing the method (24).

### MultiNicheNet analysis

*MultiNicheNet* was applied to the scRNA-seq dataset from Wilk et al. (3). *MultiNicheNet* identifies active ligand–receptor interactions between cell types of interest by taking into account not only expression of ligands and receptors themselves but also the expression of gene targets known to be regulated by those ligand–receptor pairs (25). *MultiNicheNet* was used to identify the top 50 most active ligand–receptor interactions in the dataset in which monocytes were the sender cell and NK cells were the receiver cell (using the default *MultiNicheNet* parameters). We then manually pruned the list of the top 50 interactions to remove any interactions that were erroneously included in the *NicheNet* interaction database or those that would be highly unlikely to occur between NK cells and monocytes (e.g., proteins that only interact with each other within the same cell). We manually removed 25 interactions, leaving us with 25 remaining ligand–receptor pairs. We then used *MultiNicheNet* to visualize expression of the target genes downstream of these interactions that were differentially expressed in severe COVID-19 and healthy donors. More detail regarding the detailed methodology underpinning *MultiNicheNet* can be found in the article describing the method (25).

### Quantification and statistical analysis

Flow cytometry data visualization was performed using FlowJo v10.7.1. The figures were generated in R using the *ggplot2*, *Seurat*, and *Scriabin* packages. Colors for the figures were generated using the *NatParksPalettes* package. Statistical analyses were performed as described in the figure legends and plotted using the R *ggpubr* package.

### Data and Code Availability Statement

The scRNA-seq data used in this study are available on Gene Expression Omnibus (accession no. GSE174072). Processed scRNA-seq data are hosted on the COVID-19 Cell Atlas (https://www.covid19cellatlas.org/) and are listed under “Blish Lab.” The flow cytometry and O-link data generated through this study, along with the code used to generate the figures for this study, are available on the Blish laboratory github (https://github.com/BlishLab/nk_monocyte_COVID). The flow cytometry data are also available on FlowRepository (ID FR-FCM-Z75U).

## Results

### Transcriptomic analysis reveals NK cell–monocyte cross-talk in severe COVID-19

**FIGURE 1.**
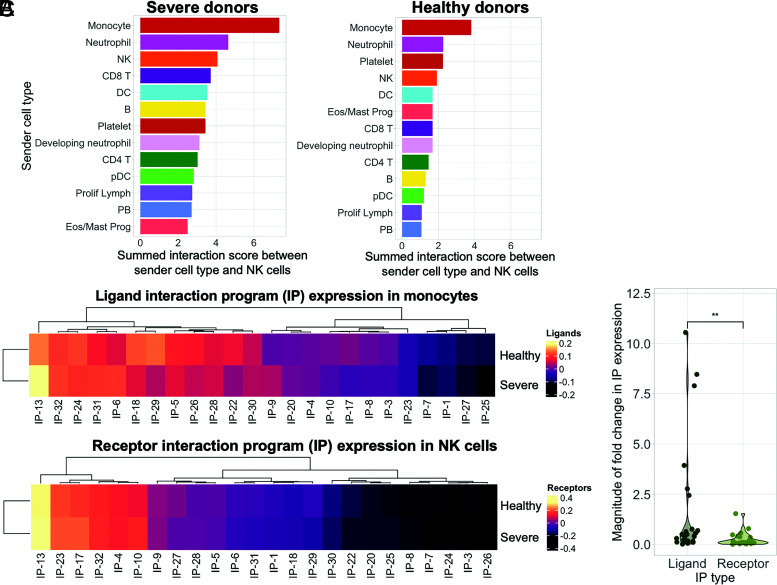
Cell–cell communication analysis reveals robust monocyte–NK cell cross-talk in severe COVID-19. A scRNA-seq dataset from Wilk et al. (3) was analyzed using the R package *Scriabin.* (**A**) Bar plots showing the summed interaction score between a given sender cell type (shown on the *y* axis) and NK cells. A larger score indicates more individual points of interaction between the sender cell type and NK cells. Interactions between cell types are shown separately from severe COVID-19 donors (*left*) and healthy control donors (*right*). (**B**) Heat maps showing the scaled expression of each interaction program (IP) that is significantly expressed in NK cells and monocytes from severe COVID-19 and healthy donors. The top heat map shows scaled expression of the ligands for each interaction program in monocytes. The bottom heat map shows scaled expression of the receptors for each interaction program in NK cells. (**C**) Violin plot showing the magnitude of fold change in expression for each IP in monocytes (Ligand) and NK cells (Receptor) between healthy and severe COVID-19 samples. Magnitudes of fold change were calculated by taking the absolute value of the fold change in expression of each IP between healthy and severe samples. Significance values were calculated using a Wilcoxon signed-rank test.

We began by identifying the cell types interacting with NK cells in severe COVID-19. To do so, we first probed the cell–cell communication pathways that may contribute to the NK cell activation, proliferation, and dysfunction in severe COVID-19 using Single-Cell Resolution Analysis through Binning (“Scriabin”) (24). We applied this method to a previously acquired COVID-19 dataset (3) to identify the interactions in which the NK cells were the receiving cell type and the cell types that were the predicted senders of these signals. To do this, we applied Scriabin’s IP discovery workflow, which identifies groups of ligands and receptors that are significantly coexpressed by the same sets of sender and receiver cells. These IPs thus represent modules of highly topologically connected cell–cell communication pathways. When generating IPs with NK cells acting as the receiver cell type, we found that monocytes had the highest coexpression of ligands (both soluble and membrane-bound) for NK cell receptors (Fig. 1A). Interestingly, even in healthy donors, NK cells received most signals from monocytes; however, the magnitude of these interactions was much greater in the setting of severe COVID-19. We therefore focused our efforts on analyzing interactions between NK cells and monocytes.

Given the strong monocyte–NK cell cross-talk suggested by this analysis, we next explored what ligand–receptor pairs underpinned these interactions, and their predicted effects on downstream NK cell transcriptional phenotype. We performed this analysis on NK cells and monocytes from healthy and severe COVID-19 donors and found 24 IPs that were significantly coexpressed by either healthy donor cells or severe COVID-19 donor cells (Supplemental Table I). Heat maps showing the scaled expression of the ligands for these programs in monocytes or the receptors for these programs in NK cells demonstrate that the bulk of the changes in severe COVID-19 patients occur in monocyte expression of ligands rather than in NK cell expression of receptors (Fig. 1B). Quantifying the magnitude of fold change in each IP between healthy and severe donors also shows that there are significantly greater changes in ligand IP expression than in receptor IP expression (Fig. 1C).

### Monocyte interactions with NK cells in severe COVID-19 are predicted to induce activation, proliferation, and apoptosis in NK cells

**FIGURE 2.**
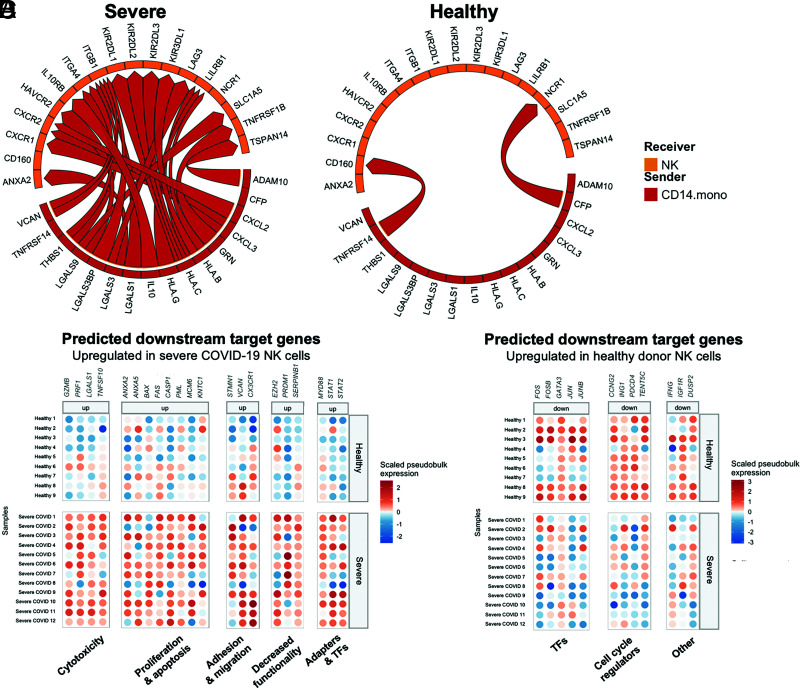
Monocyte interactions with NK cells in severe COVID-19 are predicted to induce activation, proliferation, and apoptosis in NK cells. NK cells and monocytes from severe COVID-19 and healthy donors from the scRNA-seq dataset published in Wilk et al. (3) were analyzed using the R package *MultiNicheNet*. (**A** and **B**) Circos plots showing 26 of the top ligand–receptor interaction pairs that were identified by *MultiNicheNet* as being most active between NK cells and monocytes in this dataset. Arrows point from each ligand (red; expressed on monocytes) to the corresponding receptor (orange; expressed on NK cells). (A) shows ligand–receptor pairs that are more highly expressed in severe COVID-19 patients, and (B) shows ligand–receptor pairs that are more highly expressed in healthy donors. (**C** and **D**) Bubble heat map showing measured expression in NK cells of the differentially expressed genes regulated by top ligand–receptor interactions shown in (A) and (B). Each row represents a unique donor; each column represents a unique gene. Genes were manually grouped based on function. Genes in (C) were significantly upregulated in NK cells from severe COVID-19 patients; genes in (D) were significantly upregulated in NK cells from healthy donors.

We next used the *MultiNicheNet* package (25) to identify the top 50 interaction (receptor–ligand) pairs among both severe COVID-19 and healthy donors in which monocytes were the sender cell and NK cells were the receiver cell. *MultiNicheNet* determines “top” (or high-priority) interactions by weighing a number of factors, including cell type–specific ligand and target expression levels, coexpression of ligands and receptors in the same donor, and predicted ligand activity (25). Of the top 50 interaction pairs identified by *MultiNicheNet*, we manually identified 26 whose ligand–receptor interaction had been experimentally validated and published (26–47). Twenty-four of these interaction pairs were more highly expressed in severe COVID-19 samples (Fig. 2A), whereas only two were more highly expressed in healthy donor samples (Fig. 2B), further illustrating the increased level of NK cell–monocyte cross-talk in severe COVID-19.

Among the top interactions in severe COVID-19 samples were several interactions between MHC class I molecules and killer Ig-like receptors (KIRs); interactions between the inhibitory receptors LILRB1, Tim-3 (*HAVCR2*), and Lag-3 and their cognate ligands; and interactions involving chemokine receptors and integrins (Fig. 2A). Meanwhile, the top interactions upregulated in healthy donors were the interaction between *TNFRSF14* (HVEM) and the checkpoint inhibitor CD160 and the interaction between CFP (complement factor P) and *NCR1* (activating receptor NKp46) (Fig. 2B).

To understand the effects of these interactions on the NK cell transcriptome, we used *MultiNicheNet* to visualize the expression of predicted downstream target genes in NK cells from severe COVID-19 and healthy donors (Fig. 2; Supplemental Fig. 1). This analysis revealed that interactions between monocytes and NK cells in severe COVID-19 were predicted to upregulate genes involved in NK cell cytotoxicity [*GZMB* (48), *PRF1* (49), *LGALS1* (50), *TNFSF10* (51)], proliferation [*MCM6* (52), *KNTC1* (53)], apoptosis [*ANXA2* (54), *ANXA5* (54), *BAX* (55), *FAS* (56), *CASP1* (57), *PML* (58)], cellular adhesion [*STMN1* (59), *VCAN* (35)], and migration [*CX3CR1* (60)]. Other predicted downstream targets in severe COVID-19 included several genes associated with decreased NK cell functionality [*EZH2* (61), *PRDM1* (62), *SERPINB1* (63)], two STAT family transcription factors [*STAT1*, *STAT2* (64)], and signaling adaptor *MYD88* (65) (Fig. 2C).

By contrast, the target genes downstream of the predicted monocyte–NK cell interactions in healthy donors included AP-1 family transcription factors [*FOS*, *FOSB*, *JUN*, *JUNB* (66)], as well as *GATA3* (67). Notably, monocyte–NK interactions in these samples were also predicted to upregulate several genes involved in cell cycle regulation [*CCNG2* (68), *ING1* (69), *PDCD4* (70), *TENT5C* (71)] (Fig. 2D), which contrasts with the genes involved in proliferation and apoptosis upregulated in severe COVID-19 patient NK cells (Fig. 2C). Among other upregulated targets in healthy donors were *IFNG* (72), *IGF1R* (73), and *DUSP2* (74) (Fig. 2D). Collectively, our *MultiNicheNet* analyses support the hypothesis that NK cell–monocyte interactions in severe COVID-19 may contribute to the activated and dysfunctional phenotype that has been previously described in the NK cells of severe COVID-19 patients.

### NK cells from hospitalized COVID-19 patients are activated and proliferative

**FIGURE 3.**
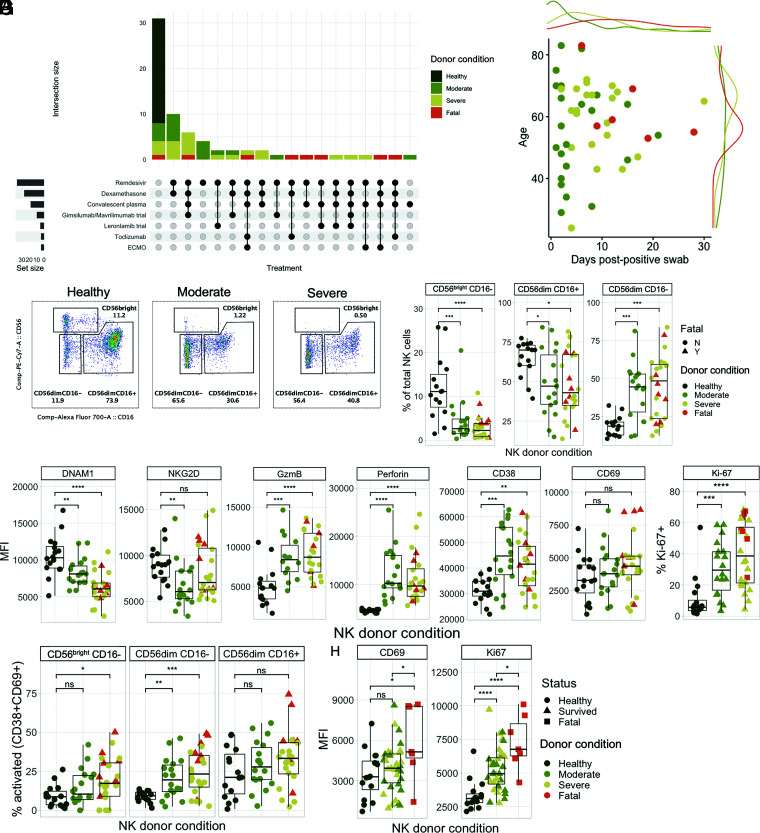
NK cells from hospitalized COVID-19 patients are phenotypically altered. (**A**) Upset plot showing the number of patients in each treatment group, colored by patient severity. (**B**) Scatter plot showing the distribution of age (*y* axis) and days postpositive test swab (*x* axis) within the cohort of COVID-19–positive donors. (**C** and **D**) Representative flow plots (C) and box plots (D) showing the proportion of classical NK cell subsets defined by expression of CD56 and CD16 in NK cells from healthy, moderate COVID (Moderate), and severe COVID (Severe) donors. (**E**) Box plots showing the mean fluorescence intensity (MFI) of activating receptors DNAM-1 and NKG2D, cytotoxic molecules Granzyme B and Perforin, and activation markers CD38 and CD69 in all NK cells from patients across severity groups. (**F**) Percentage of NK cells positive for the proliferation marker Ki-67 out of all live NK cells from patients across severity groups. (**G**) Box plots showing the proportion of activated (CD38^+^ CD69^+^) NK cells in each of the three NK cell subsets identified in subfigure 1B across severity groups. (**H**) Box plots showing expression of CD69 and Ki-67 in the NK cells of fatal COVID-19 cases compared with healthy or hospitalized, nonfatal cases. Significance values for all plots in this figure were determined using an unpaired Wilcoxon rank-sum test with the Bonferroni correction for multiple hypothesis testing.

Having identified monocytes as a cell type that interacts strongly with NK cells in COVID-19, we next sought to devise an in vitro experimental system that would allow us to interrogate NK cell–monocyte interactions using primary immune cells from COVID-19–positive donors. For this, we used a cohort of 44 hospitalized donors collected from an observational cohort study at UCLA, as well as 17 healthy donors from the Stanford Blood Bank (Fig. 3A, 3B). Given that previous studies, including by our group, have identified striking phenotypic differences in the NK cells of hospitalized COVID-19 patients compared with those of mild COVID-19 patients and healthy controls (1, 4,5, 7, 9–11, 75,76), we first sought to assess phenotypic changes in the NK cells of this cohort of COVID-19 patients. Similar to prior studies, we found that COVID-19 induces a shift in the frequency of NK cell subsets defined by expression of CD56 and CD16. NK cells from COVID-19 patients had a significant increase in the frequency of unconventional CD56^dim^ CD16^lo^ NK cells and a corresponding decrease in the frequencies of both CD56^dim^ CD16^hi^ and CD56^bright^ CD16^lo^ NK cells (Fig. 3C, 3D).

The NK cells of COVID-19 patients also exhibited changes in their expression of key surface and intracellular molecules (Fig. 3E, 3F; Supplemental Fig. 2). Surface expression of the activating receptor NKG2D and the activating coreceptor DNAM-1 were decreased in COVID-19 patients compared with healthy controls while having greatly increased intracellular expression of the cytotoxic molecules Granzyme B and Perforin and the proliferation marker Ki-67. Markers of activation CD38 and CD69 were more highly expressed on COVID-19 patient NK cells. Notably, Granzyme B and Perforin were predicted downstream targets of ligands expressed by monocytes in severe COVID-19 (Fig. 2C). These changes were generally conserved in all three NK cell subsets analyzed (CD56^bright^ CD16^lo^, CD56^dim^ CD16^lo^, and CD56^dim^ CD16^hi^) (Supplemental Fig. 3A–F). However, we found that the proportion of activated NK cells (defined as CD38^+^ CD69^+^) increased more substantially in the unconventional CD56^dim^ CD16^lo^ population than in the other subsets (Fig. 3G).

Most analyses of NK cells thus far have not had adequate numbers of fatal COVID-19 samples to assess differences between hospitalized patients who survived their disease course and those that did not. Given that our cohort contained a relatively high number of fatal COVID-19 cases (7), we assessed whether any NK cell markers were differentially expressed between fatal and nonfatal patients. Although expression of most markers was not significantly different between fatal and nonfatal cases (data not shown), we identified two markers, CD69 and Ki-67, that were more highly expressed in fatal cases compared with hospitalized but nonfatal cases (Fig. 3H).

The samples from hospitalized COVID-19 patients in this cohort were collected from 2020 to 2021, before the availability of standardized treatment recommendations or outpatient therapeutics such as nirmatrelvir/ritonavir, and the patients were therefore treated with a variety of therapeutics (Fig. 3A). To assess any potential effects of these interventions on NK cell phenotype, we compared NK cells from patients who did not receive each treatment to those who did. In general, we saw no differences in treated versus untreated groups except for those clearly correlated with disease severity (data not shown); it is difficult to disentangle effects due to treatment from those due to severity given that interventions are often administered on a severity-dependent basis. However, we did find that the NK cells from the four patients on ECMO had significantly lower expression of Perforin than those of patients not on ECMO (Supplemental Fig. 2G).

### COVID-19 patient NK cells exhibit defective killing of SARS-CoV-2–infected and bystander cells

**FIGURE 4.**
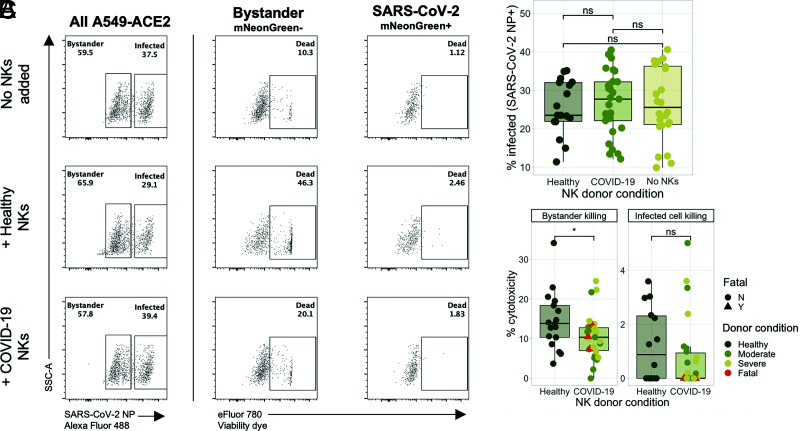
COVID-19 patient NK cells exhibit defective killing of SARS-CoV-2–infected and bystander cells. (**A**) Representative flow plots showing the percentage of infected A549-ACE2 target cells (*left*) and percentage of dead target cells (center and right) in wells with target cells only (top), healthy NK cells plus target cells (middle), and COVID-19 NK cells plus target cells (bottom). (**B**) Box plot showing the percentage of infected (SARS-CoV-2 NP^+^) target cells in wells with healthy or COVID-19^+^ NK cells versus wells with target cells only. (**C**) Box plot showing the background-subtracted killing of bystander (*left*) or SARS-CoV-2–infected (*right*) target cells after a 3-h coculture with NK cells from either healthy or COVID-19^+^ donors. An effector:target ratio of 10:1 was used. Significance values for all box plots were determined using a Wilcoxon ranked-sum test. For the comparison between healthy and COVID-19 NK killing of bystander cells, *p* = 0.042; for the comparison between healthy and COVID-19 NK killing of SARS-CoV-2–infected cells, *p* = 0.31.

Although the phenotype of severe COVID-19 patient NK cells suggests that they have high cytotoxic potential, other studies have shown that these cells respond poorly to K562 tumor target cells (1,2, 10,11). Compared to NK cells from healthy donors, COVID-19 patient NK cells are also worse at reducing viral replication when cocultured with SARS-CoV-2–infected cells (10,11). However, no study to date has directly assessed the ability of COVID-19 patient NK cells to kill SARS-CoV-2–infected cells and bystander (uninfected) cells. We previously demonstrated that NK cells are impaired in their ability to lyse SARS-CoV-2–infected target cells due to target cell loss of the ligands for the activating receptor NKG2D (77). We therefore performed killing assays as previously described (77) using NK cells isolated from either healthy donors or COVID-19 patients and A549-ACE2 target cells infected with an mNeonGreen-tagged strain of SARS-CoV-2. By infecting the target cells with an multiplicity of infection sufficient to result in ∼25% infected cells and using the mNeonGreen fluorescent tag, we were able to differentiate between direct killing of SARS-CoV-2–infected cells and bystander cells. There were no significant differences in the proportion of SARS-CoV-2 NP^+^ target cells between wells containing NK cells from healthy donors, wells containing NK cells from COVID-19^+^ donors, and wells containing target cells only (Fig. 4B). We found that COVID-19 patient NK cells were significantly worse at killing bystander A549-ACE2 cells compared with NK cells from healthy donors (Fig. 4C). Additionally, consistent with prior studies, we found that both healthy donor NK cells and COVID-19 patient NK cells killed SARS-CoV-2–infected cells less frequently than bystander cells (77,78) (Fig. 4C). We found that COVID-19 patient NK cells appeared to exhibit a disease severity–dependent defect in their ability to kill SARS-CoV-2–infected cells, although this reduction was not statistically significant (Fig. 4C).

### Monocytes from severe COVID-19 patients induce activation and proliferation in healthy NK cells

**FIGURE 5.**
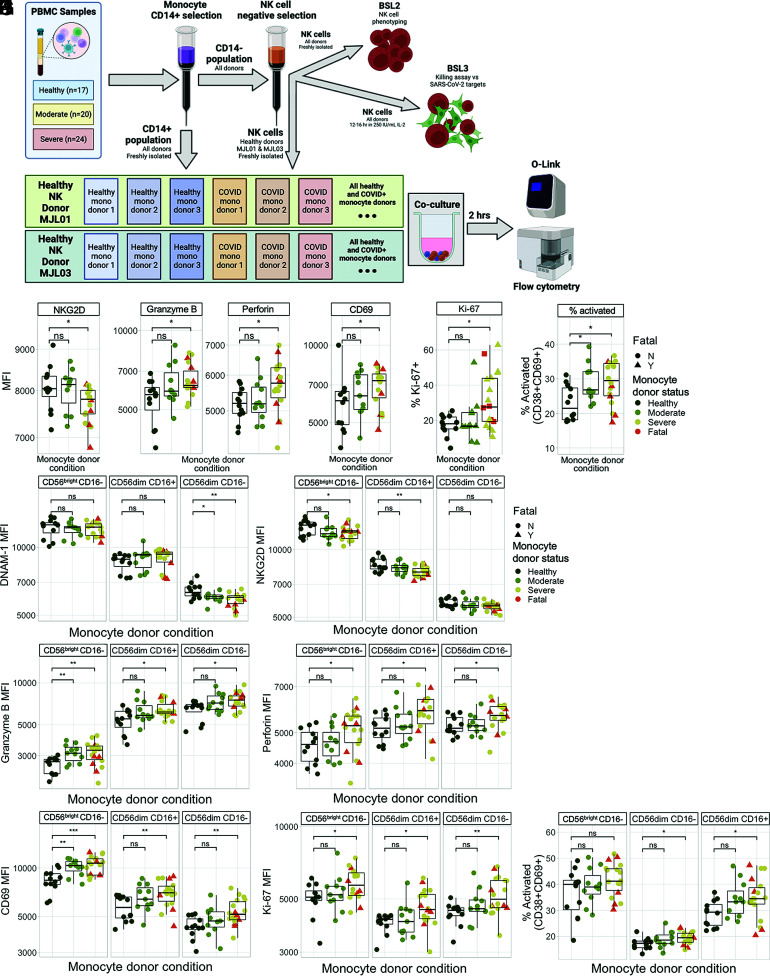
Monocytes from severe COVID-19 patients induce activation and proliferation in healthy NK cells. (**A**) Schematic illustrating the experimental design for allogeneic NK cell–monocyte cocultures. Briefly, CD14^+^ monocytes and NK cells were isolated from all donor PBMC. The NK cells from two healthy donors (MJL01 and MJL03) were cocultured with CD14^+^ monocytes from allogeneic healthy or COVID-19^+^ donors for 2 h in either a round-bottom 96-well plate or a Transwell plate. After 2 h, NK cell phenotypes were assessed via flow cytometry. (**B–E**) Box plots showing the mean fluorescence intensities (MFIs) of NKG2D (B), Granzyme B and Perforin (C), CD69 (D), and Ki-67 (E) in all NK cells from healthy donors following a 2-h coculture with allogeneic monocytes. (**F**) Box plot showing the percentage of healthy donor NK cells positive for both CD38 and CD69 following a 2-h coculture with allogeneic monocytes. (**G–J**) Box plots showing the MFIs of DNAM-1 and NKG2D (G), Granzyme B and Perforin (H), CD69 (I), and the percentage of NK cells positive for Ki-67 (J) in three distinct subsets of NK cells from healthy donors following 2-h coculture with allogeneic monocytes. (**K**) Box plots showing the percentage of healthy donor NK cells from three distinct subsets of NK cells that were positive for both CD38 and CD69 following a 2-h coculture with allogeneic monocytes. Significance values for all plots in this figure were determined using an unpaired Wilcoxon rank-sum test with the Bonferroni correction for multiple hypothesis testing.

To experimentally validate whether NK cell–monocyte interactions could indeed underlie NK cell activation in severe COVID-19, we developed an allogeneic coculture system in which CD14^+^ monocytes from COVID-19 patients were isolated and cocultured with NK cells derived from healthy donors. After 2 h of coculture, we assessed NK cell phenotype by flow cytometry to determine whether COVID-19 patient monocytes were capable of inducing changes in healthy NK cells (Fig. 5A; Supplemental Fig. 3).

We found that coculture of healthy NK cells with monocytes from severe COVID-19 patients recapitulated many of the key features of the NK cells from these same severe COVID-19 patients (shown in Fig. 4). Following coculture with monocytes from severe COVID-19 patients but not moderate patients or healthy donors, healthy NK cells had decreased expression of NKG2D (Fig. 5B). They also had increased expression of the cytotoxic molecules Granzyme B and Perforin (Fig. 5C), as well as the activation and tissue residency marker CD69 (Fig. 5D) and the proliferation marker Ki-67 (Fig. 5E). Finally, NK cells cocultured with monocytes from both moderate and severe COVID-19 patients had a higher proportion of activated (CD38^+^ CD69^+^) cells (Fig. 5F).

As observed in the NK cells of COVID-19 patients, most of the changes induced in healthy NK cells by COVID-19 patient monocytes were conserved across the three NK cell subsets examined (Fig. 5G–K). Expression of Granzyme B, Perforin, Ki-67, and CD69 was increased in CD56^bright^ CD16^lo^, CD56^dim^ CD16^lo^, and CD56^dim^ CD16^hi^ NK cells (Fig. 5H–K). However, expression of DNAM-1 and NKG2D was affected differentially in different subsets: DNAM-1 expression was only significantly decreased in unconventional CD56^dim^ CD16^lo^ cells, whereas NKG2D was only downregulated in the other two subsets (CD56^bright^ CD16^lo^ and CD56^dim^ CD16^hi^) (Fig. 5G). Together, these data indicate that monocytes from COVID-19 patients are sufficient to induce activation and proliferation of NK cells, as well as downregulation of NKG2D and DNAM-1.

### Monocyte-induced CD56^dim^ NK cell activation in severe COVID-19 occurs primarily through contact-dependent mechanisms

**FIGURE 6.**
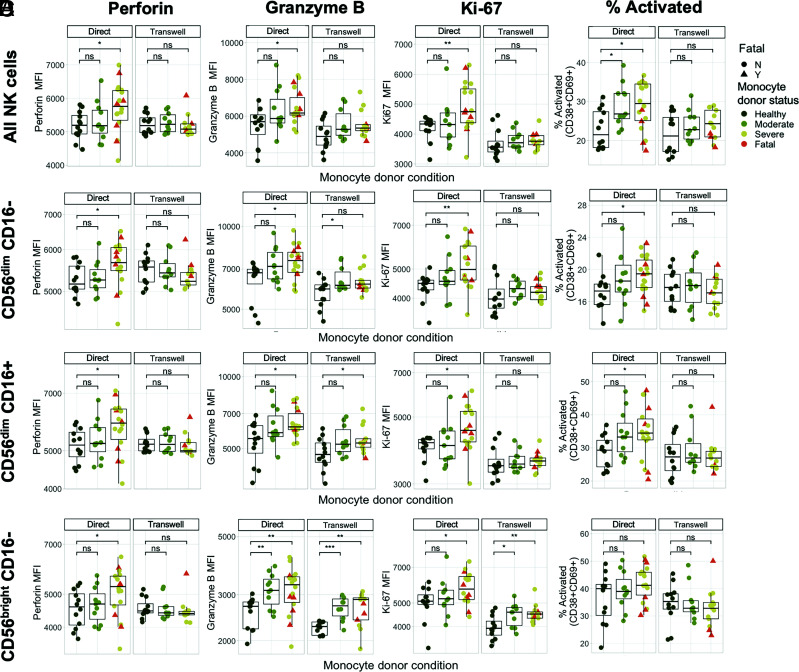
CD56^bright^ NK cells are activated by monocytes through contact-independent mechanisms, whereas CD56^dim^ NK cells are activated primarily through contact-dependent mechanisms. (**A–D**) Box plots showing the mean fluorescence intensities (MFIS) of Perforin, Granzyme B, and Ki-67 and the percentages of NK cells positive for CD38 and CD69 across severity conditions in normal (round-bottom 96-well plate) cultures and Transwell cultures. Each row shows the expression of these markers in a different population of NK cells: total NK cells (A); CD56^dim^ CD16^−^ NK cells (B); CD56^dim^ CD16^+^ NK cells (C); or CD56^bright^ CD16^−^ NK cells (D).

To determine mechanisms by which monocytes activate NK cells, we examined whether activation occurred through contact-dependent or contact-independent mechanisms. We performed our coculture experiments using either a direct culture system (as shown in Fig. 5) or a Transwell culture system with monocytes on the bottom and NK cells in a Transwell insert to abrogate direct contact. We first analyzed the total population of NK cells following either direct or Transwell coculture with monocytes (Fig. 6A; Supplemental Fig. 4A) and both CD16+ and CD16^−^ CD56^dim^ NK cells, which typically make up >95% of peripheral blood NK cells (Fig. 6B, 6C; Supplemental Fig. 4C, 4D). Although NK cells that underwent direct coculture with monocytes from severe COVID-19 patients exhibited increases in cytotoxic molecule expression, proliferation, and activation, we found that all of these statistically significant increases in marker expression were abrogated in the NK cells that instead underwent Transwell coculture (Fig. 6A; Supplemental Fig. 4). These results suggest that the activated, proliferative NK cell phenotype induced by coculture with monocytes from severe COVID-19 patients is primarily mediated by direct cell–cell contacts in CD56^dim^ NK cells, although small trending increases in Granzyme B, Ki-67, and coexpression of CD38 and CD69 imply that there may be roles for soluble factors in this activation as well.

### CD56^bright^ NK cells are activated through soluble interactions with monocytes from severe COVID-19 patients

Although CD56^dim^ NK cells were only minimally activated in a Transwell setting, we found that CD56^bright^ CD16^−^ NK cells had strongly increased expression of Granzyme B and Ki-67 after Transwell coculture with monocytes from severe COVID-19 patients (Fig. 6D), although these cells did not undergo an increase in Perforin expression in a Transwell setting (Fig. 6D). These results suggest that the mechanisms by which monocytes from severe COVID-19 patients activate NK cells differ between subsets of NK cells, with CD56^bright^ CD16^−^ cells being more strongly affected by soluble factors and CD56^dim^ NK cells being more strongly affected by direct cell–cell interactions.

### Proinflammatory and prochemotactic cytokines contribute to the NK cell phenotype

**FIGURE 7.**
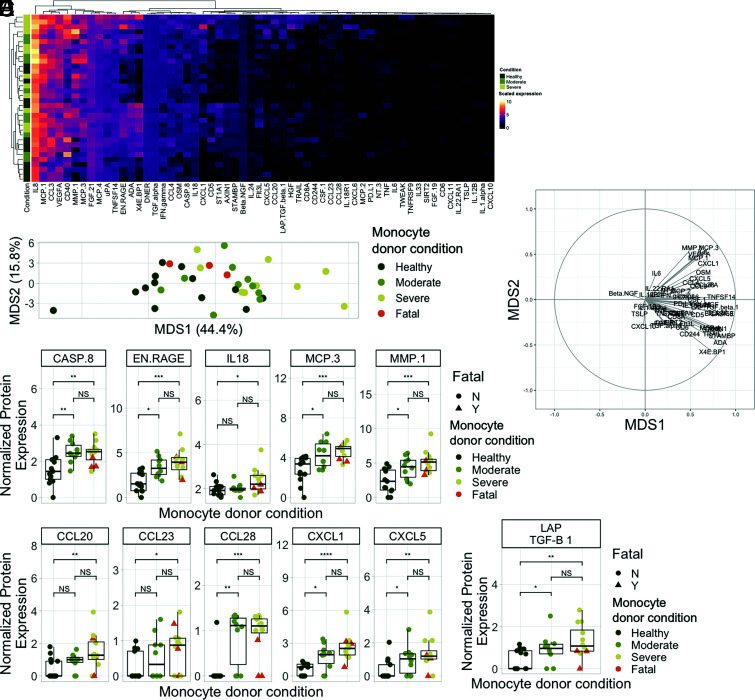
Monocytes from COVID-19 patients induce an inflammatory and prochemotactic cytokine environment. (**A**) Heat map showing normalized protein expression of the 58 analytes from the O-link inflammation panel that were detected in at least one sample. (**B**) Multidimensional scaling (MDS) plot. (**C**) Circle plot. (**D–F**) Box plots showing the normalized protein expression of various analytes in the O-link inflammation panel across monocyte donor severity conditions.

We next sought to identify soluble factors that may have induced activation of CD56^bright^ CD16^−^ NK cells in the presence of monocytes from severe COVID-19 patients. We performed O-link analysis using the inflammation panel to assay 92 soluble markers including cytokines, chemokines, and growth factors on the supernatants from our Transwell coculture experiments. Due to limited cell numbers, we were not able to evaluate monocytes in the absence of NK cells. Of the 92 analytes measured, 58 were detected in at least one sample measured (Fig. 7A). We performed multidimensional scaling analysis using the 58 markers detected, revealing almost complete separation between severe COVID-19 patient samples and healthy donors, with moderate COVID-19 patient samples occupying an intermediate space (Fig. 7B). Interestingly, the three fatal donor samples included in this analysis clustered more closely with the healthy donor samples than with the severe, nonfatal donor samples (Fig. 7B). The separation between severe and healthy donors in multidimensional space was driven by increased expression of a wide variety of analytes in the severe patient samples, including MMP-1, MCP-3, CXCL5, IL-18, CXCL1, VEG-F-A, and Latency-associated peptide TGF-β1 (LAP TGF B1) (Fig. 7C).

Interrogating expression of individual markers, we found five notable proinflammatory analytes (Caspase-8, EN-RAGE, IL-18, MCP-3, and MMP-1) that were present at higher concentrations in the supernatants of severe COVID-19 patient monocyte cultures compared with healthy monocyte cultures (Fig. 7D). Cultures containing monocytes from severe COVID-19 patients also had significantly elevated levels of many chemokines that are known to regulate the immune response to infectious disease (Fig. 7E). In addition to the observed increase in expression of inflammatory analytes and chemokines, we also found a severity-dependent increase in LAP TGF-β1 (Fig. 7F), which is used as a biomarker for active TGF-β1 (79,80). TGF-β1 itself could not be accurately measured in these samples because active TGF-β1 detection assays cross-react with the FBS present in the cell culture medium (80). As previously noted, high levels of TGF-β1 in the serum of severe COVID-19 patients have been shown to induce NK cell dysfunction in COVID-19 (10).

## Discussion

Although it has been well established that peripheral NK cells from severe COVID-19 patients take on a unique phenotype and are highly dysfunctional, the mechanisms underlying these changes were not previously well understood. Other studies have suggested that this phenotype may partially result from type I IFN signaling (4, 10,11), and TGF-β in the serum of COVID-19 patients has been shown to have an inhibitory effect on NK cells (10). Cross-talk between NK cells and other immune cells is a key regulator of the NK cell response in other infections (15,16, 20, 81,82), leading us to interrogate how NK cell interactions with other cell types underlie NK cell activation and dysfunction in COVID-19. Here, we performed computational analyses showing that NK cells interact strongly with monocytes in severe COVID-19. We then validated experimentally that monocytes from severe COVID-19 patients can induce an activated phenotype in healthy NK cells, similar to the phenotype observed in primary NK cells from severe COVID-19 patients. We further demonstrated that these interactions occur via both contact-dependent and contact-independent mechanisms. Our results collectively illustrate the importance of NK cell communication with other peripheral immune cells in severe COVID-19 and explore a novel mechanism of NK cell activation in this setting.

All three major subsets of NK cells analyzed (CD56^bright^ CD16^−^, CD56^dim^ CD16^−^, and CD56^dim^ CD16^+^) underwent significant phenotypic changes following direct coculture with monocytes from severe COVID-19 patients. However, the mechanisms underlying the changes in each of these subsets appear to differ; nearly all of the changes in CD56^dim^ NK cells following direct coculture were abrogated when the coculture was performed in a Transwell setting, whereas CD56^bright^ NK cells were activated in both Transwell and direct settings. These results imply that CD56^dim^ NK cells are activated primarily through contact-dependent mechanisms. Indeed, results from our previous work suggested that NK cells may receive activation through the expression of ligands for NKG2D and DNAM-1 on monocytes in severe COVID-19 patients (3). This hypothesis is further supported by the fact that downregulation of DNAM-1 on CD56^dim^ CD16^−^ NK cells and NKG2D on CD56^dim^ CD16^+^ NK cells was only observed in direct and not Transwell cultures in this study (Supplemental Fig. 4). Both NKG2D and DNAM-1 can be internalized following ligation, leading to loss of expression on the cell surface (83,84). Our use of the recently developed computational technique Scriabin to infer cell–cell communication between NK cells and other peripheral immune cells at the single-cell level also provided candidate contact-dependent interactions that may underlie the activation and dysfunction of CD56^dim^ NK cells by monocytes in severe COVID-19. Notably, many of the interactions predicted by Scriabin to be upregulated in severe COVID-19 are interactions between class I MHC molecules and KIR or LILRB family receptors, which typically have an inhibitory effect on NK cell cytotoxicity (85,86). The activating and inhibitory signals received by CD56^dim^ NK cells from monocytes in severe COVID-19 may therefore explain these cells’ activated yet dysfunctional state.

Although CD56^dim^ NK cell activation by monocytes was largely abrogated in Transwell cocultures, Granzyme B and Ki-67 in CD56^bright^ NK cells were strongly upregulated in Transwell cocultures (Fig. 6B). A plausible explanation for this observation is the fact that CD56^bright^ NK cells typically express much higher levels of cytokine and chemokine receptors than their CD56^dim^ counterparts and may therefore experience stronger activation by the suite of proinflammatory mediators present in the supernatants of cultures containing severe COVID-19 patient monocytes. Three of these analytes (Caspase-8, EN-RAGE, and IL-18) are all associated with the NLRP3 inflammasome (87–90). In addition to its role in inducing cell death, Caspase-8 also has a noncytotoxic role in modulating NK and CD8^+^ T cell responses to viral infection (91). RAGE, the receptor for EN-RAGE (S100A12), is expressed on human (92) and murine (93) NK cells. S100A8/9 have been shown to activate NK cells through RAGE (93), although the ability of S100A12 to activate NK cells through RAGE has not yet been directly assessed. Monocyte chemoattractant protein 3 (MCP-3), also known as CCL7, induces not only lymphocyte chemotaxis but also NK cell activation (94). CD56^bright^ NK cells express increased levels of the receptors for MCP-1 (95,96) and IL-18R (97,98), as well as TGF-β receptor 2 and TGF-β receptor 3 (99). Conversely, CD56^dim^ NK cells express higher levels of the KIRs, whose contact-dependent interactions with MHC class I on monocytes were identified by Scriabin as being significantly upregulated in severe COVID-19 (Fig. 2A, 2B). One important caveat of the results of our Transwell experiments is that the physical separation in the Transwell system and therefore lower local concentrations of cytokines may cause or enhance the abrogation in NK cell activation that we see in these cocultures compared with direct cultures. Hence, we cannot rule out that the cytokines involved in CD56^bright^ NK cell activation by COVID-19 patient monocytes may also play a role in CD56^dim^ NK cell activation when present in higher local concentrations.

The results of this study generate new insights into NK cell and monocyte trafficking in severe COVID-19. We found that cocultures of severe COVID-19 patient monocytes and healthy NK cells had significantly increased concentrations of multiple chemokines compared with cocultures of healthy donor monocytes with healthy NK cells. The upregulated chemokines include CCL23 (100), CCL28 (101), CXCL1 (102), and CXCL5 (103), all of which are involved in monocyte chemotaxis. CCL23 also stimulates secretion of matrix metalloproteinases in monocytes (100). CCL20 is not chemoattractive to monocytes but does induce chemotaxis in NK cells and other lymphocytes (104,105). Notably, the cells used in these experiments were derived from blood rather than from the site of infection in the airways; therefore, the role of these chemokines in recruiting immune cells to participate in the immune response to SARS-CoV-2 in this setting is unclear. However, in addition to examining chemokine secretion, we also assessed expression of CD69 on NK cells, which is a marker of tissue residency (106) and actively aids NK cell retention in tissue (107). CD69 is upregulated on the NK cells of severe COVID-19 patients (3, 5, 10, 108,109) and was significantly upregulated following coculture of healthy NK cells with severe COVID-19 patient monocytes in our study (Fig. 5B). NK cells have also been shown to traffic to the site of infection in severe COVID-19 (7, 12–14, 95). Therefore, interactions between NK cells and monocytes in the periphery may drive CD69 expression that leads to NK cell retention in the lungs during severe COVID-19.

This study has several limitations that should be noted when interpreting its results. Cryopreserved PBMCs were used rather than fresh, which can affect cell phenotype and functionality (110,111); this also raises the possibility that the differences in NK cell phenotype and function observed in COVID-19 patient NK cells could be due to differential survival of COVID-19 NK cell populations during freezing and thawing. However, other studies that used fresh PBMCs found similar patterns of activation and dysfunction in COVID-19 patient NK cells (3, 5), making this explanation unlikely. We were also only able to coculture NK cells and monocytes for 2 h, which may have limited the phenotypic changes that we could observe in NK cells.

Activation of NK cells by monocytes has been documented in several other infectious diseases, but overall, relatively little is known about monocyte–NK cell cross-talk in viral infection in comparison with NK cell communication with cell subsets such as dendritic cells and T cells (81). Most work thus far has focused on the ability of NK cells to lyse virally infected monocytes and macrophages (19, 112,113). The small body of work examining cross-talk between NK cells and uninfected monocytes has centered around monocyte secretion of cytokines that are known to modulate NK cell responses, including IL-12 (114) and IL-18 (112). Our work here describes both soluble and contact-dependent interactions that take place between NK cells and uninfected monocytes away from the site of infection and influence NK cell phenotype. These insights further our understanding of immune polyphony in the setting of infectious disease, which in turn informs the designs of critical vaccines and therapeutics seeking to modulate the immune response.

## Supplementary Material

Supplemental 1 (PDF)
