# Supplementary material for: NK Cell–Monocyte Cross-talk Underlies NK Cell Activation in Severe COVID-19
Source: J Immunol. 2024 Apr 26;212(11):1693–705. doi: 10.4049/jimmunol.2300731 (PMC11102029; doi:10.4049/jimmunol.2300731)
Supplement: Supplemental 1 (PDF) [file JI_2300731_Supplemental_1.pdf]

IP-1

- DLL1=NOTCH3
- EFNA1=EPHB1
- ERBB2=ERBB3
- IGF2=IGF1R
- IGF2=IGF2R
- IL12A=IL12RB1
- IL17A=IL17RA
- IL17A=IL17RC
- IL25=IL17RB
- NCAM1=FGFR1
- RLN2=RXFP2
- SEMA7A=PLXNC1
- XCL1=XCR1

IP-10

- CALCB=RAMP1
- COL18A1=GPC1
- FGF5=FGFR1
- GDF6=BMPR1A
- GDF6=BMPR2

IP-13

- ADAM17=NOTCH1
- ADAM9=ITGAV
- ALCAM=CD6
- BAG6=NCR3
- CXCL16=CXCR6
- ICAM1=ITGAL
- ICAM1=ITGAM
- ICAM1=ITGB2
- IL15=IL2RB
- IL15=IL2RG
- INSR=ADRB2
- JAG1=NOTCH1
- LGALS9=HAVCR2
- NRG1=ERBB2
- PVR=CD226
- SIRPA=CD47
- TGFB1=TGFBR3
- TIMP1=CD63

IP-17

- ANGPT1=TIE1
- APOE=LRP8
- APOE=VLDLR
- BMP4=BMPR1A
- BMP4=BMPR1B
- BMP4=BMPR2
- GDF9=BMPR1A
- IL23A=IL12RB1
- NCR3LG1=NCR3
- POMC=MC1R

IP-18

- CCL2=CCR5
- CCL3=CCR5
- CCL3L3=CCR5
- IFNL2=IL10RB
- NPW=NPBWR1
- PLG=F2RL1
- TIMP2=ITGA3

IP-20

- CCL8=CCR1
- CCL8=CCR2
- GAL=GALR2
- INHBB=ACVR1B
- INHBB=ACVR2A

IP-22

- ADAM17=ERBB4
- DLL3=NOTCH1
- DLL3=NOTCH2
- DLL3=NOTCH3
- DLL3=NOTCH4
- ERBB2=ERBB4
- EREG=ERBB4
- HBEGF=ERBB4
- IFNW1=IFNAR1
- IL13=IL13RA1
- IL13=IL4R
- IL17F=IL17RA
- NRG1=ERBB4
- NRG2=ERBB3
- NRG4=ERBB4

IP-23

- BMP2=ACVR2A
- BMP2=ACVR2B
- BMP2=BMPR1A
- BMP2=BMPR1B
- BMP2=BMPR2
- COL1A1=DDR2
- EGFR=ERBB2
- IL12A=IL12RB1
- PDGFD=PDGFRB

IP-25

- APOE=LDLR
- APOE=LRP1
- CCL4=CCR1
- CCL4=CCR5
- CCL5=CCR1
- CD244=CD48
- DLL1=NOTCH2
- FASLG=FAS
- GDF11=ACVR2B
- IFNG=IFNGR1
- IFNG=IFNGR2
- IL16=CD4
- JAG2=NOTCH2
- JAG2=NOTCH3
- LTA=TNFRSF14
- LTA=TNFRSF1A
- LTA=TNFRSF1B
- TF=TFRC
- TGFB1=TGFBR2
- TGFB3=TGFBR2
- TGFBR1=TGFBR2
- TNFSF14=TNFRSF14
- TRAF2=TNFRSF1B
- VEGFB=FLT1

IP-27

- CCL5=CCR5
- DLL1=NOTCH1
- DLL1=NOTCH2
- F2=F2RL1
- F2=THBD
- FAT4=DCHS1
- GDF11=ACVR2B
- IGF2=IGF1R
- IGF2=IGF2R
- IL5=CSF2RB
- JAG2=NOTCH1
- LAMA2=RPSA
- TGFB3=TGFBR1
- VEGFB=NRP1
- WNT1=RYK
- WNT3=FZD8

IP-24

- ADCYAP1=VIPR1
- ADM=CALCRL
- ANXA1=FPR1
- APOB=LDLR
- APP=CD74
- APP=LRP1
- CCL14=CCR1
- CCL14=CCR5
- CCL2=CCR1
- CCL2=CCR2
- CCL3=CCR1
- CCL3L3=CCR1
- CCL5=CCR1
- CSF1=CSF1R
- CTF1=IL6ST
- EDN1=EDNRB
- EFNB1=EPHB2
- ERBB3=NRG1
- F12=CD93
- GAS6=MERTK
- HBEGF=CD9
- ICAM1=ITGAM
- ICAM1=ITGB2
- IGF2=INSR
- IL10=IL10RB
- IL15=IL15RA
- IL1A=IL1RAP
- IL1B=IL1R1
- IL1B=IL1R2
- IL1B=IL1RAP
- IL1RAP=IL1R1
- IL1RN=IL1R1
- IL1RN=IL1R2
- IL6=IL6R
- IL6=IL6ST
- IL6R=IL6ST
- JAG1=NOTCH2
- JAG1=NOTCH4
- LIFR=IL6ST
- LPL=LRP1
- MST1=MST1R
- NTS=NTSR1
- OSM=IL6ST
- PGF=FLT1
- PLAT=LRP1
- PTPRF=INSR
- SELPLG=SELL
- SEMA3C=NRP2
- SEMA3C=PLXND1
- SEMA3E=PLXND1
- SEMA4A=PLXND1
- SERPINA1=LRP1
- SHH=PTCH2
- TF=TFRC
- THBS1=CD36
- THBS1=LRP1
- TIMP1=CD63
- TNF=TNFRSF1A
- TNF=TNFRSF1B
- TNFSF10=TNFRSF10A
- TNFSF10=TNFRSF10B
- TNFSF10=TNFRSF10D
- TNFSF12=TNFRSF12A
- TNFSF14=LTBR
- VCAN=CD44
- VEGFA=FLT1

IP-26

- A2M=LRP1
- ADAM10=NOTCH2
- ANXA1=FPR1
- ANXA1=FPR2
- APP=TNFRSF21
- B2M=CD1A
- BMPR2=ACVR1
- CALR=LRP1
- CCL2=CCR5
- CCL28=CCR3
- CCL3=CCR5
- CCL3L3=CCR5
- CCL4=CCR1
- CCL5=CCR3
- CD244=CD48
- CD47=SIRPA
- CD6=ALCAM
- DLL1=NOTCH3
- EFNA1=EPHB1
- EFNA4=EPHA2
- EFNA5=EPHA2
- EFNA5=EPHB2
- EFNB1=EPHB2
- EFNB1=EPHB3
- F2=F2RL1
- F2=THBD
- FGFR1=CDH1
- GAS6=AXL
- HLA-A=LILRB1
- HLA-A=LILRB2
- HLA-B=LILRB2
- HLA-G=LILRB1
- HLA-G=LILRB2
- IFNG=IFNGR2
- IGF2=INSR
- IL16=CD4
- INSL3=RXFP2
- ITGAL=ICAM1
- JAG1=NOTCH3
- LTA=LTBR
- PTPRF=INSR
- RSPO1=LGR4
- SELPLG=SELL
- SEMA3C=NRP2
- SEMA4D=PLXNB2
- SEMA7A=PLXNC1
- SERPINC1=LRP1
- TGFB1=ACVRL1
- TGFB1=APP
- TGFB1=SDC2
- TGFB3=ACVRL1
- TNF=TNFRSF1A
- TNF=TNFRSF1B
- TNFSF12=TNFRSF1
- TNFSF14=LTBR
- TRAF2=TNFRSF1B
- VEGFA=NRP1
- VEGFB=NRP1
- XCL1=XCR1

IP-28

- CCL13=ACKR4
- CD274=PDCD1
- CXCL10=CXCR3
- CXCL11=CXCR3
- CXCL9=CXCR3
- EFNA4=EPHA6
- EFNB2=EPHB1
- IL17B=IL17RB
- IL19=IL20RB
- IL22=IL10RB
- PDCD1LG2=PDCD1
- TNFSF13=TNFRSF13
- TNFSF13B=TNFRSF
- TNFSF13B=TNFRSF
- WNT3A=FZD6

IP-29

- CXCL1=CXCR1
- CXCL1=CXCR2
- CXCL2=CXCR1
- CXCL2=CXCR2
- CXCL3=CXCR1
- CXCL3=CXCR2
- CXCL5=CXCR1
- CXCL5=CXCR2
- CXCL6=CXCR1
- CXCL6=CXCR2
- EFNB2=EPHB4
- FGF22=FGFR2
- FGF23=FGFR2
- FGF5=FGFR2
- IL1A=IL1R1
- IL1B=IL1R1
- IL1RN=IL1R1
- MMP9=CD44
- PPBP=CXCR1
- PPBP=CXCR2
- SPP1=CD44

IP-3

- CD28=CD86
- CD40LG=CD40
- FLT3LG=FLT3
- SEMA3A=NRP1
- WNT7A=FZD5

IP-30

- CCL13=CCR3
- CCL8=CCR5
- CSF2=CSF2RA
- CSF2=CSF2RB
- CSF2=CSF3R
- EFNB1=EPHB1
- GAS6=AXL
- IL22=IL10RB
- INSR=CEACAM1
- NRG1=ERBB3
- SEMA3E=PLXND1
- TAC1=TACR2
- WNT3A=FZD2
- WNT3A=FZD5
- WNT3A=FZD7
- WNT3A=RYK

IP-31

- ADAM9=ITGAV
- CALCA=CALCRL
- CD28=CD86
- CD40LG=CD40
- CD86=CD28
- CD86=CTLA4
- CRH=CRHR1
- CSF1=CSF1R
- CTLA4=CD86
- DKK1=KREMEN1
- EPO=EPOR
- FBN1=ITGAV
- FBN1=ITGB1
- FN1=ITGA4
- FN1=ITGA5
- FN1=ITGAV
- FN1=ITGB1
- ICAM1=ITGAL
- ICOS=ICOSLG
- ICOSLG=ICOS
- IL10=IL10RA
- IL10=IL10RB
- IL27=IL27RA
- INHBB=ACVR1B
- INHBB=ACVR2A
- INSL3=RXFP2
- INSR=ADRB2
- JAG1=NOTCH1
- LGALS9=HAVCR2
- MMP9=CD44
- NRG1=ERBB3
- PCSK9=LDLR
- PRL=PRLR
- SEMA3C=PLXND1
- SIRPA=CD47
- SPP1=CD44
- THBS1=CD47
- TNFSF15=TNFRSF25
- WNT3A=FZD8

IP-5

- APP=TNFRSF21
- EFNA1=EPHA2
- EFNA4=EPHA2
- EFNA5=EPHA2
- FGFR1=CDH1
- VEGFA=NRP1

IP-6

- EPHB2=EFNB2
- F12=GP1BA
- IL1A=IL1R1
- IL1A=IL1R2
- IL1A=IL1RAP
- SELPLG=SELP

IP-9

- CALR=ITGA3
- DLL4=NOTCH1
- DLL4=NOTCH2
- DLL4=NOTCH3
- DLL4=NOTCH4
- GDF5=ACVR2B
- GDF5=BMPR1A
- GDF5=BMPR1B
- GDF5=BMPR2
- GDF9=BMPR1A
- IL11=IL11RA
- IL11=IL6ST
- INHA=TGFBR3
- LHB=LHCGR
- NODAL=ACVR2B
- RARRES2=CMKLR1
- TIMP2=ITGA3

IP-32

- ADAM17=NOTCH1
- ALCAM=CD6
- APOE=VLDLR
- BAG6=NCR3
- BMP7=ACVR1
- BMP7=ACVR2A
- BMP7=ACVR2B
- BMP7=BMPR1A
- BMP7=BMPR1B
- BMP7=BMPR2
- BMPR2=BMPR1B
- CALCB=RAMP1
- CD48=CD244
- CD58=CD2
- CDH1=PTPRM
- CXCL16=CXCR6
- EFNA4=EPHA4
- EFNA5=EPHA4
- EFNB1=EPHB3
- EFNB2=EPHA4
- EFNB2=EPHB2
- EFNB2=EPHB3
- EPHB2=EFNB2
- F12=CD93
- GAS6=MERTK
- HLA-A=KIR3DL2
- HLA-B=KIR3DL1
- HLA-C=KIR2DL1
- HLA-C=KIR2DL3
- HLA-E=KLRC2
- HLA-E=KLRD1
- ICAM3=ITGAL
- IHH=PTCH1
- IL15=IL2RB
- IL15=IL2RG
- IL18=IL18R1
- IL4=IL13RA1
- IL4=IL2RG
- IL4=IL4R
- IL4R=IL2RG
- IL6=IL6R
- IL6=IL6ST
- IL7=IL2RG
- ITGB1BP1=ITGB1
- JAG1=NOTCH3
- LIF=IL6ST
- MADCAM1=ITGA4
- MADCAM1=ITGB7
- MICA=KLRK1
- NCR3LG1=NCR3
- NOTCH1=DLL1
- NRG1=ERBB2
- PDGFD=PDGFRB
- PLAU=PLAUR
- POMC=MC1R
- PTPRJ=PDGFRB
- PVR=CD226
- RARRES2=CMKLR1
- RELN=LRP8
- RELN=VLDLR
- SHH=PTCH1
- TGFB1=TGFBR1
- TGFB1=TGFBR2
- TGFB1=TGFBR3
- TGFB2=TGFBR1
- TGFB2=TGFBR2
- TGFB2=TGFBR3
- TGFB3=TGFBR1

IP-4

- BMP4=BMPR1A
- BMP4=BMPR1B
- BMP4=BMPR2
- BMP6=BMPR1A
- BMP6=BMPR1B
- BMP6=BMPR2
- CCL8=CCR5
- CDH1=ITGAE
- CDH1=PTPRM
- CX3CL1=CX3CR1
- DLK1=NOTCH1
- EGF=ERBB2
- FBN1=ITGAV
- FBN1=ITGB1
- FGF2=FGFR1
- FGF2=FGFRL1
- FGF2=SDC2
- FGF2=SDC3
- FGF2=SDC4
- FGF23=FGFR1
- IHH=PTCH1
- IL4=IL13RA1
- IL4=IL2RG
- IL4=IL4R
- PDGFB=PDGFRB
- RSPO1=LGR6
- SELP=SELPLG
- TGFB2=TGFBR1
- TGFB2=TGFBR2
- TGFB2=TGFBR3
- TNFSF18=TNFRSF18
- WNT4=FZD6

IP-7

- A2M=LRP1
- CD6=ALCAM
- JAG2=NOTCH3
- LTA=LTBR
- LTA=TNFRSF14
- LTA=TNFRSF1A
- LTA=TNFRSF1B
- PLG=F2RL1
- SEMA4D=PLXNB1
- TGFB3=ACVRL1

IP-8

- APOE=LDLR
- APOE=LRP1
- APOE=LRP8
- BGN=TLR2
- BGN=TLR4
- CALCB=CALCRL
- CCL16=CCR1
- CCL16=CCR2
- CCL23=CCR1
- CCL8=CCR1
- CCL8=CCR2
- EDN3=EDNRB
- EFNA5=EPHB2
- EFNB1=EPHB1
- F9=LRP1
- ICAM3=ITGB2
- OSM=IL6ST
- TGFB1=SDC2

Supplementary Table 1: Ligand-receptor pairs defining each interaction program. Table showing the ligand-receptor pairs assigned to each interaction program (“IP”) by Scriabin. Ligand-receptor pairs are shown as “ligand”=“receptor”. Each box shows the ligand-receptor pairs that comprise one interaction program.

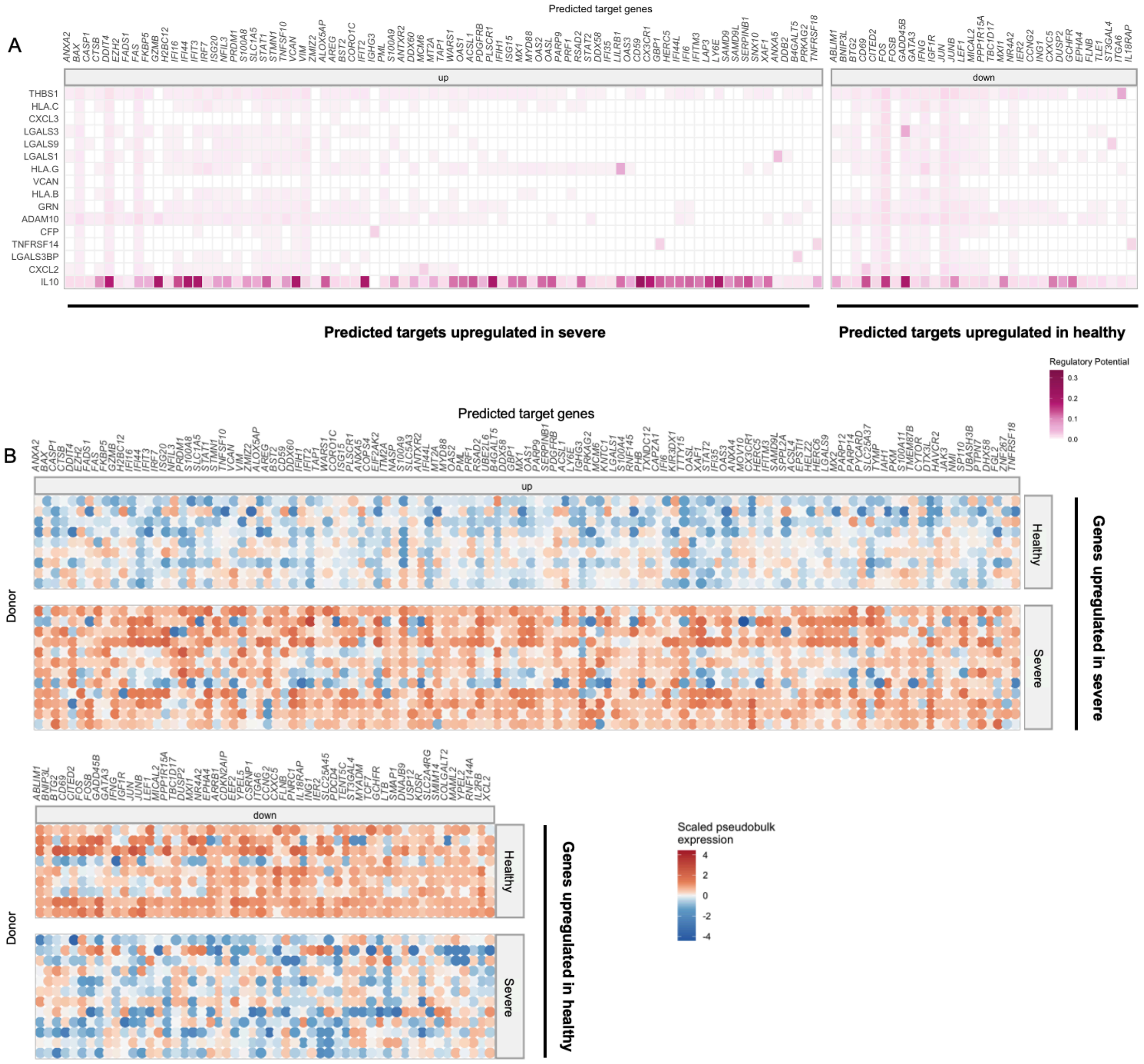

Supplementary Figure 1: MultiNicheNet analysis of predicted ligand-receptor interactions and downstream gene expression. *MultiNicheNet* was applied to the NK cells and monocytes from severe COVID-19 and healthy donors from the data set Wilk et al. 2020 in order to identify the most active ligand-receptor interaction pairs in each donor condition and to assess expression of the genes downstream of those predicted interaction pairs. A) Heatmap showing regulation of differentially expressed target genes by the top ligand-receptor pairs connecting NK cells and monocytes. Ligands (expressed on monocytes) are listed on the y-axis. Each column represents one gene that is differentially expressed between severe and healthy donors (left block: higher in severe; right block: higher in healthy). Box color illustrates the potential for each ligand to regulate each gene. B) Bubble heatmap showing measured expression in NK cells of the differentially expressed genes regulated by top ligand-receptor interactions. Each row represents a unique donor; each column represents a unique gene.

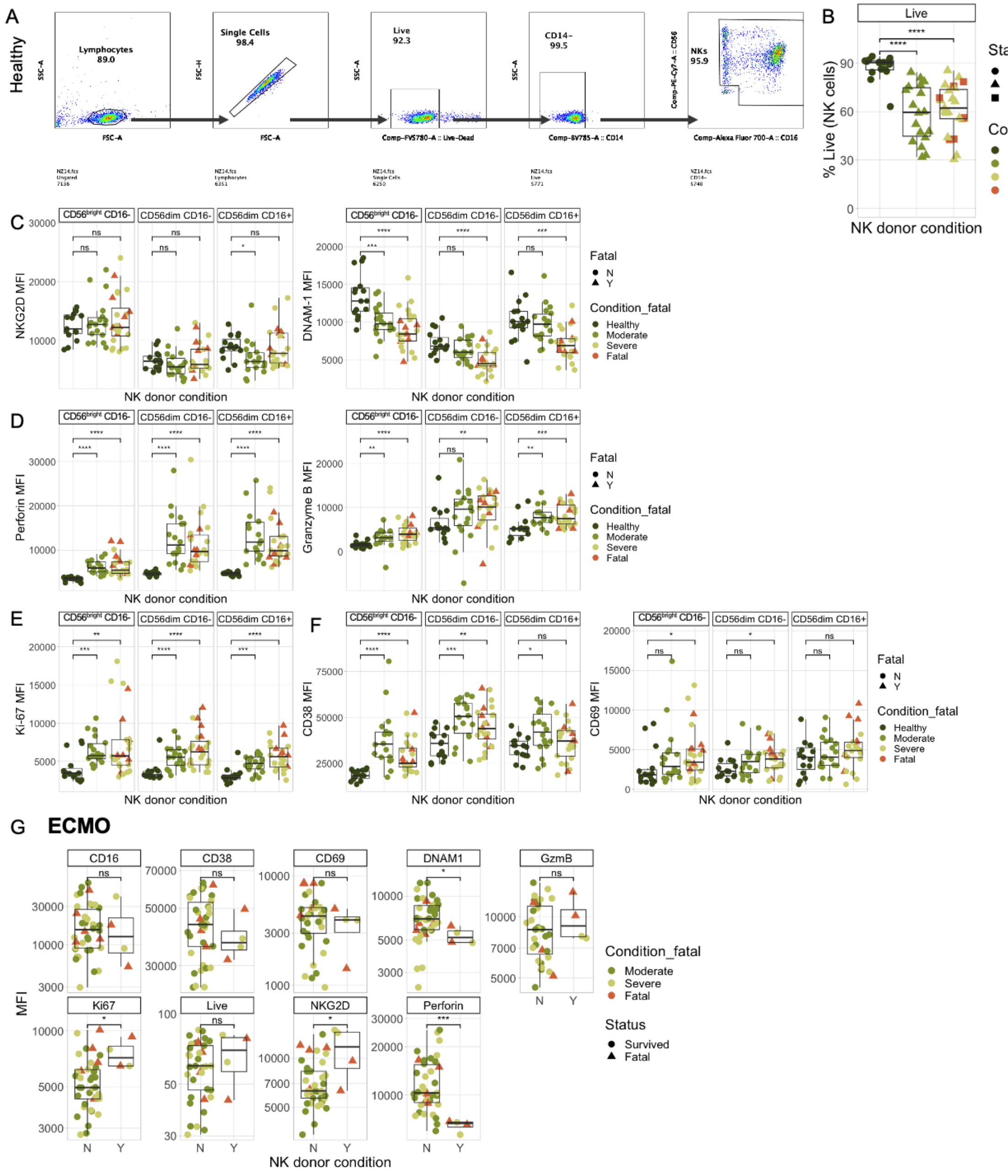

Supplementary Figure 2: Gating strategy and additional analyses for primary NK cell phenotyping experiments. A) Representative flow plots showing the gating strategy used to identify NK cells in NK cell phenotyping experiments. Example plots are shown for a healthy donor. B) Boxplot showing the percentage of NK cells that were live (negative for eFluor 780 fixable viability dye) in each sample. C-F) Boxplots showing the MFIs of each marker shown in main text Fig. 2 across three major NK cell subsets ($CD56^{+}CD16^{-}$, left; $CD56^{dim}CD16^{-}$, center; $CD56^{dim}CD16^{+}$, right) from healthy or COVID-19+ donors. G) Boxplots showing MFIs of all markers in NK cells in COVID-19 patients who were treated ("Y") or not treated ("N") with extracorporeal membrane oxygenation ("ECMO"). Statistical significance values for all plots were determined using a Wilcoxon rank sum test.

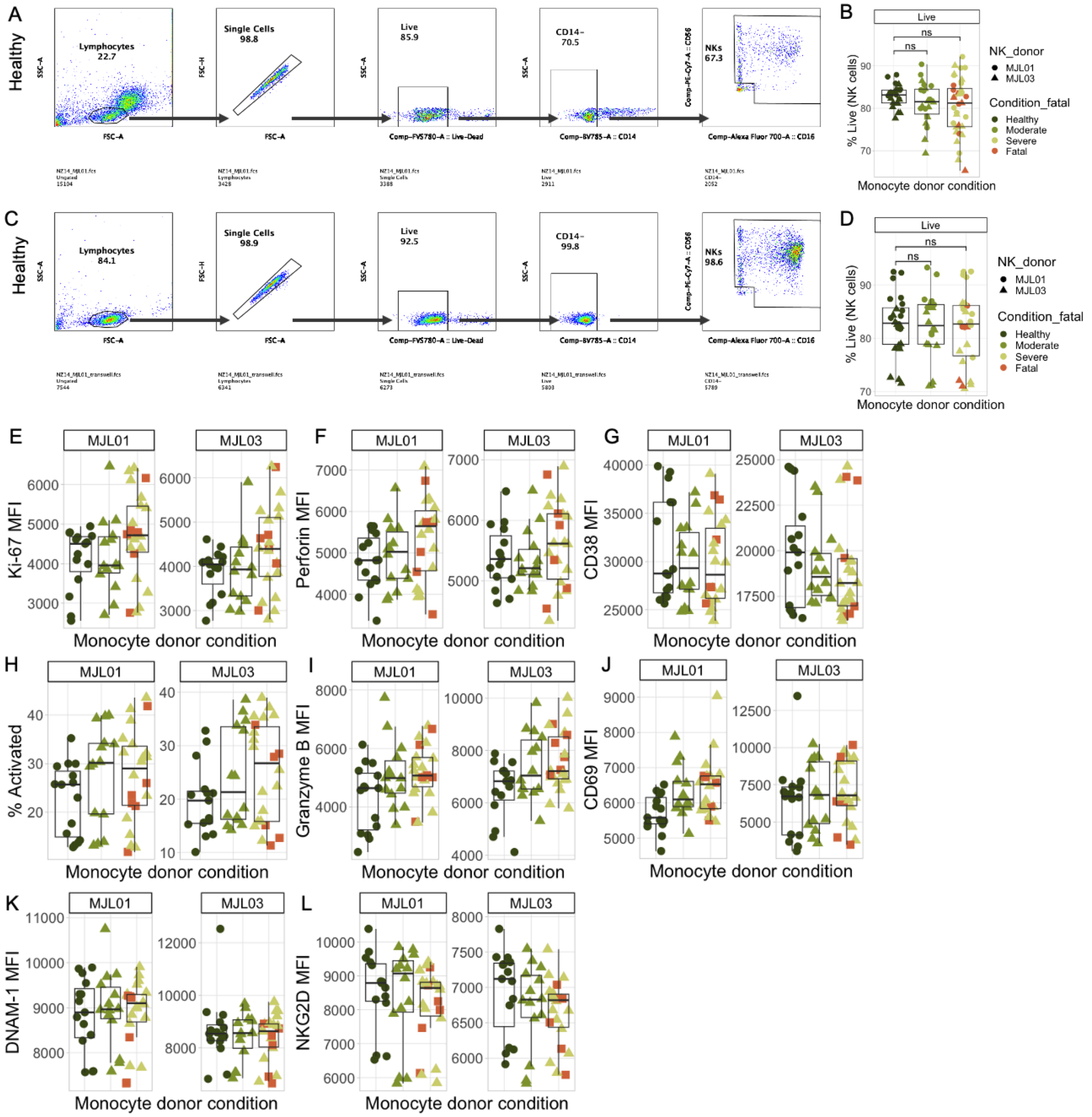

Supplementary Figure 3: Gating strategies and individual healthy NK cell donor analyses for NK-monocyte co-culture experiments. A) Representative flow plots showing the gating strategy used to identify NK cells in direct NK cell-monocyte co-culture experiments. Example plots are shown for a healthy donor. B) Boxplot showing the percentage of NK cells that were live (negative for eFluor 780 fixable viability dye) in each sample following direct co-culture. C) Representative flow plots showing the gating strategy used to identify NK cells in transwell NK cell-monocyte co-culture experiments. Example plots are shown for a healthy donor. D) Boxplot showing the percentage of NK cells that were live (negative for eFluor 780 fixable viability dye) in each sample following transwell co-culture. E-L) Boxplots showing expression of all markers in the two healthy NK cell donors used for all co-culture experiments following 2-hour co-culture with monocytes from healthy or COVID-19+ donors.

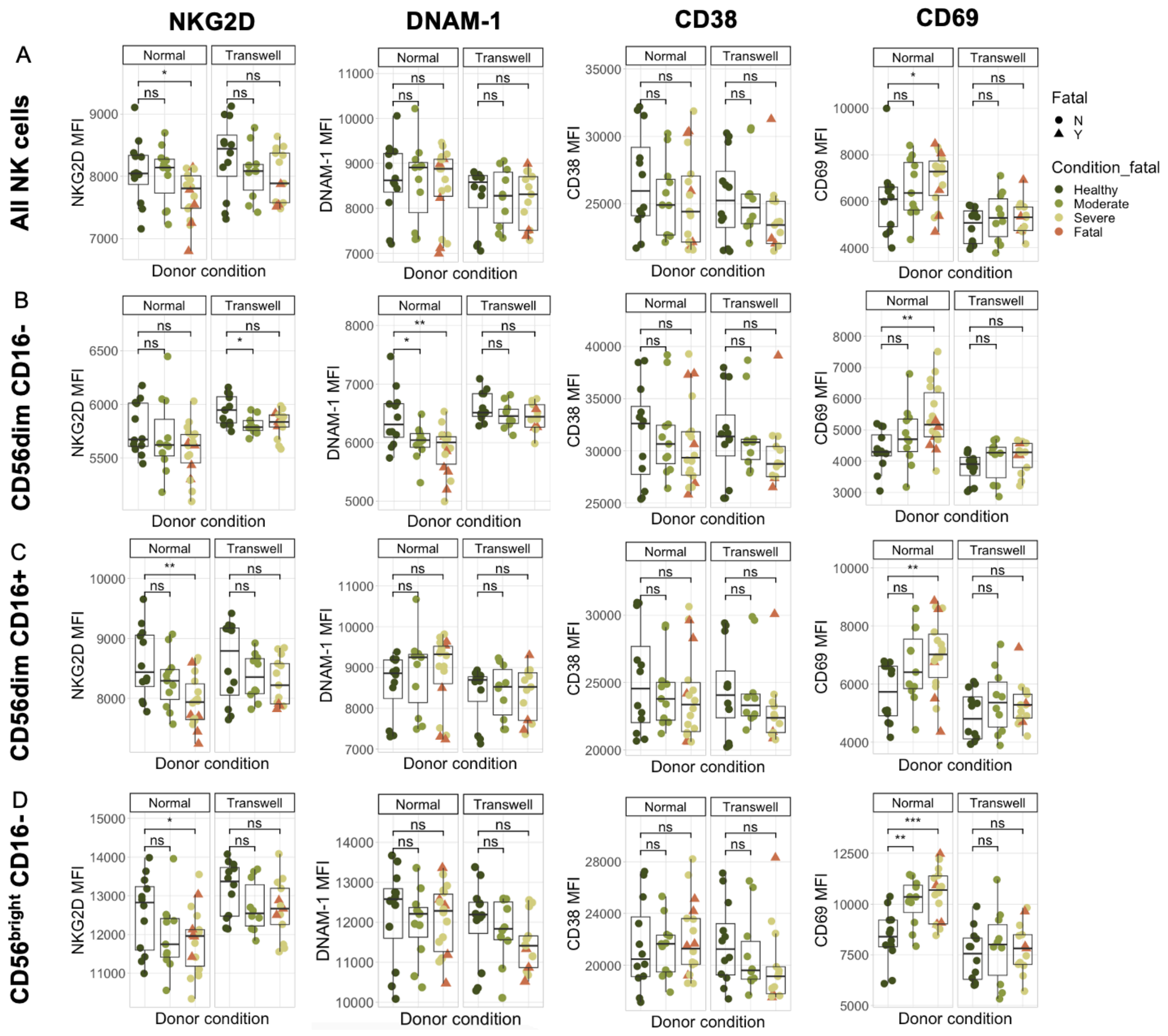

Supplementary Figure 4: Boxplots showing expression of NKG2D, DNAM-1, CD38, and CD69 in transwell vs direct co-cultures. A-D) Boxplots showing the MFI of NKG2D, DNAM-1, CD38, and CD69 across severity conditions in normal (round-bottom 96 well plate) cultures and transwell cultures. Each row shows the expression of these markers in a different population of NK cells: total NK cells (A); $CD56^{bright}$ $CD16^{-}$ NK cells (B); $CD56^{dim}$ $CD16^{-}$ NK cells (C); or $CD56^{dim}$ $CD16^{+}$ NK cells (D). Statistical significance values were determined using a Wilcoxon rank sum test with the Bonferroni correction for multiple hypothesis testing.
